# I-BaR: integrated balance rehabilitation framework

**DOI:** 10.3389/fnbot.2024.1401931

**Published:** 2024-07-03

**Authors:** Tugce Ersoy, Pınar Kaya, Elif Hocaoglu, Ramazan Unal

**Affiliations:** ^1^Department of Mechanical Engineering, Human-Centered Design Laboratory, Ozyegin University, Istanbul, Türkiye; ^2^Department of Physiotherapy and Rehabilitation, Istanbul Medipol University, Istanbul, Türkiye; ^3^Department of Electrical and Electronics Engineering, Living Robotics Laboratory, Istanbul Medipol University, Istanbul, Türkiye; ^4^SABITA (Research Institute for Health Sciences and Technologies), Istanbul Medipol University, Istanbul, Türkiye

**Keywords:** balance rehabilitation, multi-modal sensory feedback, robotic rehabilitation, anticipatory postural adjustment, compensatory postural adjustment, rehabilitation methodology

## Abstract

Neurological diseases are observed in approximately 1 billion people worldwide. A further increase is foreseen at the global level as a result of population growth and aging. Individuals with neurological disorders often experience cognitive, motor, sensory, and lower extremity dysfunctions. Thus, the possibility of falling and balance problems arise due to the postural control deficiencies that occur as a result of the deterioration in the integration of multi-sensory information. We propose a novel rehabilitation framework, Integrated Balance Rehabilitation (I-BaR), to improve the effectiveness of the rehabilitation with objective assessment, individualized therapy, convenience with different disability levels and adoption of assist-as-needed paradigm and, with integrated rehabilitation process as whole, that is, ankle-foot preparation, balance, and stepping phases, respectively. Integrated Balance Rehabilitation allows patients to improve their balance ability by providing multi-modal feedback: visual via utilization of virtual reality; vestibular via anteroposterior and mediolateral perturbations with the robotic platform; proprioceptive via haptic feedback.

## 1 Introduction

Neurological disorders impact approximately 1 billion individuals worldwide, representing a diverse range of socio-economic statuses, age groups, and ethnicities. Furthermore, an estimated 6.8 million people die each year as a result of neurological illnesses (Fineberg et al., 2013; Pehlivan et al., 2016; Who, 2022). The number of deaths from neurological disorders and disability has increased dramatically in the last 30 years, particularly in low- and middle-income countries; a further increase is foreseen worldwide due to growth in population and aging. Neurological disorders are predicted to impacting for approximately 10% of the global burden of disease and 25% of years lived with disability (Who, 2022). Along with the cost of care for neurological disorders, the financial loss related to unemployment was estimated to be $9 billion per year in Canada in 2007 (Gaskin et al., 2017) and approximately €1135 million in the United Kingdom in 2010 (Fineberg et al., 2013). In the United States, it was estimated that expenses only for stroke alone exceeded $33.6 billion in 2015 (Pehlivan et al., 2016). To provide adequate service in this regard, it is vital to take action to meet the growing demand.

Individuals with neurological disorders experience walking and movement restrictions, balance, motor control, exhaustion, and other health problems, which all directly impact their quality of life (QoL) due to impairments in the central and peripheral nervous systems (CNS and PNS). The nature and severity of the condition may differ from patient to patient depending on the lesional region at the affected CNS and PNS. In multiple sclerosis (MS), commands are conveyed through the nerve slowly or cannot be transmitted due to degeneration of the myelin sheath (Mehravar et al., 2015; Doty et al., 2018). In Parkinson's disease (PD), depigmentation of the substantia nigra and damage to dopamine-producing cells result in deficiencies in the balance control (Deng et al., 2018). Furthermore, in stroke disease, focal neurological function losses occur due to infarction or bleeding in the relevant part of the brain (Hankey, 2017). The CNS integrates information from the visual, vestibular, proprioceptive and cognitive systems, and PNS through continuous sensory re-weighting and provides postural control in static and dynamic situations (Horak, 2006). This integration of multi-sensory information is disrupted in neurological diseases, causing balance deficits and an increase in fall rate (Rito et al., 2021). More than 75% of MS patients have symptoms of poor balance. Furthermore, approximately 60% of MS patients reported at least one fall in the previous 3 months, and more than 80% reported impairments in activities of daily living (ADL) (Aruin et al., 2015a; Craig et al., 2019). It has been reported that approximately 73% of stroke patients and 45–68% of Parkinson's patients experience at least one fall per year (De Angelis et al., 2021). The factors mentioned above lead to a sedentary lifestyle and accordingly deteriorate patients' social health, which is a subdomain of QoL. This lifestyle may also lead to additional health-related issues such as obesity, diabetes, and heart disease (Craig et al., 2019; Schilling et al., 2019), which further dramatically deteriorates their QoL (Verghese et al., 2010). Due to the factors mentioned above, the search for improvements in existing rehabilitation methods continues.

With this aim, we identify the necessity of integrated balance rehabilitation (I-BaR) framework to assess and improve ankle-foot proprioception, postural control, and stepping characteristics in neurological diseases. In particular, this framework is composed of three main phases, that is, ankle-foot/preparation, balance, and stepping rehabilitation. At first, in the ankle-foot/preparation phase, the sensation of the sole, joint proprioception, and movement improvements are aimed. Second, in the balance phase, sensory weighting skills are aimed to be improved by using multi-modal feedback via perturbations. Finally, stepping rehabilitation aims to improve walking parameters via step-taking activities to target points with adjustable distances.

## 2 Methods

The skill of maintaining balance involves multiple factors that combine both physical and sensory elements. The current balance rehabilitation program uses different sequences of training, feedback, assistance, instruction, and focus of attention, as well as exercise physiology principles. These devices/tests have been developed for different disease types and severity levels. However, since these devices/tests address specific severity levels, they cannot be used in the entire rehabilitation process. To the best of our knowledge, there is no methodology developed for ankle, balance, and step-taking rehabilitation for improving postural adjustment strategies (motor learning) in patients with different disease severity. The following section presents the current finding and solution of the overview of postural control mechanisms, robot-aided rehabilitation, and their design, respectively.

### 2.1 Overview of postural control mechanisms

The somatosensory function includes the senses of touch, vibration, pressure, proprioception, pain, and temperature. Impairments in this function negatively affect the ability to perceive, distinguish, and recognize the senses in the body (Aries et al., 2021). Consequently, disorientation accompanied by abnormal movements, balance disorders, muscle weakness, and inability to maintain postural control (stabilize the body against gravity and perturbation) may occur (Kim and Jang, 2021). For instance, it is reported that post-stroke individuals experience high rates of somatosensory impairment, ranging between 65% and 85% (Costantino et al., 2017). A research with self-questionnaire demonstrated that individuals with somatosensory and motor impairments suffer from lower walking capacities and lower levels of independence in ADL (Patel et al., 2000; Tyson et al., 2013; Gorst et al., 2019). Since restoring walking ability is a primary objective for many stroke patients, establishing the best treatments for balance, gait, and mobility were identified as one of the top ten stroke research priorities (Sánchez-Blanco et al., 1999). In a recent survey conducted with 145 stroke individuals, 43% of individuals reported decreased sensation in their feet; sensory impairment was indicated to be the second most common foot problem after the loss of strength (Bowen et al., 2016; Gorst et al., 2016). Limitations in walking, high fall rate, and impairments in foot-ground contact and sense of foot position sense and hence decrease the outdoor activities in the community.

#### 2.1.1 Balance control mechanisms

Balance control, according to Shumway-Cook and Woollacott (Anne Shumway-Cook, 2016), is highly activity-specific and falls into three categories:

- *Static/dynamic steady-state balance control* is sustaining a stable posture while sitting, standing, or walking.
- *Proactive balance control* is activated before the predicted perturbation. The CNS uses postural adjustments, that is, anticipatory and compensatory postural adjustments (APAs/CPAs), envisaged as a muscular adjustment mechanism to provide balance control while maintaining body balance and vertical posture during different conditions. These adjustments engage and activate the trunk and lower extremity postural muscles before an impending external/internal perturbation occurs. It reduces the risk of balance deterioration by regulating the body's center of mass (CoM) position.
- *Reactive balance control* is activated after the perturbation to compensate CoM deviation (Granacher et al., 2011; Lesinski et al., 2015). The CNS uses CPAs as a muscular adjustment mechanism to provide this balance control. These adjustments are triggered by the sensory control signal and allow the CoM to be repositioned once it is disturbed (Aruin et al., 2015a).

After people lose their balance, they only have a few seconds to coordinate and stabilize their posture (Horak, 2006; Aruin et al., 2017). Postural perturbations such as sliding and tripping in everyday situations vary widely and are highly unpredictable. Recently, it has been proposed that studying the processes of APAs and CPAs may reveal vital information about postural control and falls. Numerous research findings show that due to postural deficiencies, falls occur during ADL (Krishnan et al., 2012; Shadmehr and Amiri, 2012; Tajali et al., 2018). The duration and magnitude of muscular activation were measured in different studies, and significant APA deficiencies were identified. There is evidence that improvements in the production of APAs can be achieved even after a single training session in individuals with stroke (Aruin et al., 2017). In another study (S. Aruin, 2016) with elderly healthy individuals shows that APAs improvement can also be achieved after four weeks of external perturbation training. These studies show that individuals exposed to predicted perturbations provide better compensatory activity in the muscles (improvement in APAs and CPAs) and more adequate body pressure center changes with the use and production of strong APAs (S. Aruin, 2016; Aruin et al., 2017). After training, early muscle activation and reduced CoM excursions took place which is substantial evidence that retraining of APAs is possible. These results form an essential basis for investigating training effectiveness focused on improving long-term APAs, CPAs, and reaction time in increasing individuals' postural control. Furthermore, they provide a background for the development of perturbation programs to improve postural control, balance, and prevent falls.

Reaction time is commonly considered in clinical diagnosis, treatment, and follow-up stages to determine the severity of postural control deficiency (APAs and CPAs) in somatosensory-based motor and neurological disorders (Saito et al., 2014; Sandroff et al., 2015). It is the time elapsed between the onset of a stimulus and when the patient's response to that stimulus begins which is physiologically divided into five parts. These are (1) seeing the stimulus at the receptor level, (2) transmitting the stimulus to the CNS, (3) transmitting the stimulus through the nerves, (4) generating the effector signal, and (5) transporting the signal to the muscles through the CNS for the mechanical work to be done (Dejanovic and Dejanovic, 2015; Agrbas et al., 2019; Tajali et al., 2019). When they examined the surface electromyography (sEMG) activity of the lower extremity muscles and the center of pressure (CoP) of both falling and non-falling stroke patients, it was found that the falling group had lower muscular electrical activity. Therefore, they need longer reaction times to prepare the posture and initiate movements through APAs (Santos et al., 2010).

The reactive and proactive balance control are managed by the activation of different neurological mechanisms of the CNS. Impairment in one of the postural adjustments (APAs and CPAs), and their ability to affect each other negatively that highlights the importance of assessing and training them in rehabilitation. Loss of balance may occur due to an unpredictable external force or failure of balance control after external/internal perturbation, that is, fast and voluntary extremities movement. Therefore, it is possible to observe improvements in both postural adjustments (proactive and reactive balance control) with effective fall prevention training (Aruin et al., 2015b, 2017; Yamada and Shinya, 2021).

A novel approach to analyze APAs employs Fitts' law to explain the relationship between APAs parameters, the length of the step, and the size of the stepping target. It is a valuable method for various target-directed movements and quantifying stability control factors. Thus, previous literature on able-bodied individuals report that Fitts' law is a valid way to explain the time to complete the foot-reaching task and APAs levels (Bertucco and Cesari, 2010; Bertucco et al., 2013; Mulder and Van Maanen, 2013; Aloraini et al., 2020). A voluntary step initiation is a self-perturbation of balance with a change in the base of support and the transition from a static to a dynamic posture, so the velocity and accuracy of movement can be assessed with Fitts' tasks since coordinated muscle activation prior to voluntary movement (APAs) is utilized to maintain the posture. A Choice Stepping Reaction Time (CSRT) test is a simple activity that evaluates person's ability to immediately trigger and execute a step with Fitts' law. The subject must step on one of the numerous targets put in front of or around them as rapidly as feasible. The time it takes to attain the goals is a promising strategy for assessing fall risk among the elderly population since they have a significantly longer duration in reaction time than non-fallers. Furthermore, a few studies investigated the inverse proportion between speed and accuracy control in foot-reaching tasks. Patients were asked to use their affected leg to step to targets with different sizes and at varying distances during these tasks (Barr et al., 2014; Tajali et al., 2019; Yamada and Shinya, 2021). However, these target points only include a switch button to calculate the reaction time. In other words, they cannot measure ground reaction force (GRF) and give haptic feedback to the patient. Yet, as neurological patients report decreased sensation on the sole and trouble in weight shifting, so it is vital to measure GRF for appropriate feedback and address aforementioned challenges in the training (Chien et al., 2017).

#### 2.1.2 The effects of perturbation-based balance training

Perturbation-based balance training (PBT) is a type of exercise in which participants are intentionally disturbed to improve reactive balance reactions by training the individual neuromuscular responses (Mansfield et al., 2015; Allin et al., 2020; Barzideh et al., 2020). This training requires performing rapidly occurring sequential whole-body movements and applying large and sudden disruptive forces to stabilize CoM (Pai et al., 2014). With the development of balance reactions, an increase in the ability to respond to the loss of balance in unpredictable ADL and consequently a decrease in fall rate can be achieved (Mansfield et al., 2015).

In addition to the physiotherapist's manual pushes and pulls (lean and release test) in PBT studies, treadmill acceleration-deceleration and inclined/moving platforms have been implemented in recent years to mimic external perturbations in daily life (Bhatt and Pai, 2008, 2009; Pai et al., 2014). It is suggested that perturbation training while walking could be an effective way to minimize fall rates in elderly people (Pai and Bhatt, 2007; Gerards et al., 2017). Current research suggests that CPAs in the elderly can be improved using PBT and that these improvements can be sustained for up to a full year after training (Gerards et al., 2017). In another study, it is shown that only a single session of perturbation is sufficient to provide permanent improvements in reactive balance control and prevent falls in elderly individuals (Aruin et al., 2017). However, to the best of our knowledge, no study has been conducted on the optimal dosage of perturbation training to induce permanent changes in reactive balance control.

Although the PBT is an approach to decrease the fall rate, it is still far from the realistic condition. The limited type of perturbations performed with existing devices and techniques may reduce individuals' capacity of adapting and generalizing the effects of PBT training to ADL. On top of that different perturbation modalities in PBT programs can be considered highly important to train balance reactions to match with a variety of situations and motor tasks (Mansfield et al., 2011; Tanvi et al., 2012; Aviles et al., 2020).

#### 2.1.3 Proprioceptive/sensorimotor training

Sensory-motor training gradually improves an individual's ability to re-weight and integrate sensory inputs to control balance and prevent falls in different somatosensory input situations (Gandolfi et al., 2015). According to these approaches, new technological devices and paradigms are being developed to promote neurorehabilitation from the CNS to the PNS. Moreover, the participation of cognitive functions increased by integrating multi-sensory feedback; thus, it improve rehabilitation effectiveness (Kearney et al., 2019; Morone et al., 2019; Verna et al., 2020).

Smania et al. (2008) show significant improvements in the ability of stroke patients to maintain balance control with a unique training program based on weight transfer and balance exercises performed under different manipulation of sensory inputs. Derakhshanfar et al. (2021) report that exteroceptive and proprioceptive stimulations, which include sensory intervention, are effective in improving motor function and ADL. In these studies, it is shown that the neuromotor system can be activated correctly by providing a change in the sensory inputs to muscle and joint receptors as well as the skin receptors of patients' feet (Kiper et al., 2015). Lim (2019) reports that a multi-sensory training program significantly improves proprioception and balance ability in patients; however, these types of studies are very limited.

The main goal of rehabilitation is the recovery of lost motor skills permanently and as quickly as possible. The effective way of training in motor learning can go through optimization of given tasks and feedback by variable sensory inputs such as pressure, vibration, and proprioception. These inputs not only facilitate motor learning but also develop compensatory mechanisms and strategies to overcome the loss of motor function resulting from a damaged neuromuscular system (Sigrist et al., 2013). Feedback (visual, auditory, or tactile) is shown to improve complex motor learning. However, in daily life, multi-modal stimuli are more dominant than uni-modal stimuli since they provide convenience in ADL. Healthy individuals process stimuli in different modalities simultaneously. Multi-modal stimuli enable the learning of several aspects of a movement simultaneously. Certain advantages of each modality are exploited, such as the ability of visualizations to show spatial aspects, and audio or tactile feedback to show temporal aspects. Moreover, it is reported that this sensory enhancement facilitates the transition between the senses (using other sense when one sense is inadequate). Humans tend to prefer multi-modal interaction as tasks become more complex, indicating an adaptive management of cognitive resources. Due to the fact that high cognitive workload in one modality can be alleviated by using another modality, enhancing motor learning (Ruffaldi et al., 2011; Shmuelof et al., 2012).

The researchers hypothesize that after training with multi-modal stimuli, sense processing would be active even when only uni-modal stimuli are present. For example, the learning process of motion detection tasks progressed positively even when auditory feedback was canceled after training with audio-visual feedback. This shows the importance of multi-modal training for complex motion recovery even at a further level (Sigrist et al., 2013; Pan et al., 2019; Morone et al., 2021). A study examined the recognition of object recognition defined by auditory and visual features. Twenty-four subjects identified objects faster by 64 milliseconds and more accurately when both feedback are combined (Giard and Peronnet (1999)). In another study, the effectiveness of multi-modal and uni-modal stimuli is compared using visual and tactile feedback. The reaction time of the participant is faster in bimodal stimuli (252.8 ms) than to single stimuli (267.8 ms) (Forster et al. (2002)). In addition, selecting the right feedback type is also important. For example, auditory feedback proved effective for learning rhythmic patterns, while visual feedback did not help with learning inter-limb coordination (Ronsse et al. (2010)).

In recent years, rehabilitation strategies that include the active participation of patients and task-oriented exercises during rehabilitation sessions are carried out by virtual reality (VR). It is integrated to increase the attention and motivation of individuals with the desire for reward and success by giving continuous feedback (Massetti et al., 2016). There are two different approaches to use VR in rehabilitation. The first one is called serious games, and they are specially designed for the rehabilitation robot/method. The second is called exercise games and refers to the use of games that already exist for entertainment purposes in rehabilitation (Gon calves et al., 2014). With the integration of robotic rehabilitation in the VR environment, it is aimed to provide visual feedback about the results of the movements performed during the rehabilitation process, increase awareness about the quality of the movements, and accelerate motor learning (Maggio et al., 2019). By including haptic cues during these applications, gait parameters such as symmetry, balance, and muscle activation patterns can be improved collectively. Thus, it is observed that the motor and cognitive status of patients can be improved at high rates by providing multi-sensory feedback and repetition of tasks with the robotic device, encouraging patients to actively participate in rehabilitation (Feys and Straudi, 2019; Maggio et al., 2019). It is reported that robotic therapy incorporated with a VR environment can assist in increasing ankle muscular strength, gait, and climbing speed (Girone et al., 2000; Boian et al., 2003; Deutsch et al., 2003; Cioi et al., 2011). The study by (Gon calves et al. (2014)) explores the use of a 1-DoF robotic platform to facilitate dorsiflexion and plantar flexion movements in post-stroke patients. The result of the study showed that muscle strength, improved actuator control, and enhanced sensory-actuator coordination significantly increased, thus improving walking patterns. Similarly, the study by Saglia et al. (2009) explores using a 2-DoF robotic platform for 4 weeks, and the results show that participants exhibited improvements in ankle proprioception.

A systematic review and meta-analysis are conducted to investigate the effectiveness of vibrotactile feedback (VF) on balance and gait rehabilitation. It shows that haptic feedback could represent a helpful intervention (De Angelis et al., 2021) which is generally divided into two types; kinesthetic and tactile. The former cue usually contains a sense of force and provides the user with a spatial frame of reference (Van Breda et al., 2017; Verna et al., 2020), while the latter cue usually includes a sense of vibration, texture, or pressure. Such feedback can be delivered via existing interfaces, which offer to the user kinesthetic and tactile sensations (Hocaoglu, 2019; Scotto di Luzio et al., 2020). When paired with other feedback, that is, kinesthetic (Afzal et al., 2018) with visual (Lee et al., 2015), the beneficial effects of VF on gait and balance parameters are found to be stronger. Because of these characteristics, VF can be employed as a supportive sensory stimulus in the context of a rehabilitation intervention focusing on sensorimotor integration. VF is also shown to be therapeutic for patients with neurological disorders (Meyer et al., 2016; Otis et al., 2016).

The interdependence of sensory, cognitive, and motor processes, as well as the need for integrated training are increasingly employed in therapies to improve ADL, motor skills and balance, in the meantime to decrease fear of falling. In other words, APAs and CPAs (proactive and reactive control adjustments) can be retrained to improve QoL.

### 2.2 Robot-aided rehabilitation

Robot-assisted rehabilitation is continuously gaining prominence as it provides more efficient training and objective evaluation than conventional rehabilitation procedures (Saglia et al., 2009, 2010; Shakti et al., 2018). Furthermore, conventional methods require at least three physiotherapists to support the lower extremity and trunk of the patient manually. Another limitation is that the effectiveness of the rehabilitation during these practices depends on the personal knowledge and experience of the therapist. It is reported that the demand for physiotherapists is increasing continuously to match the number of patients due to the increase in the aging population worldwide. The use of robotic devices to address these challenges is encouraged to shift the adoption of rehabilitation clinics from conventional methods to robotic-assisted rehabilitation. Hence, high-quality therapy sessions can be achieved at a relatively low cost and with significantly less effort (Díaz et al., 2011; Krebs and Volpe, 2013; Yurkewich et al., 2015; Kalita et al., 2021).

Most neurological patients have a reduced range of motion (ROM) in their ankle as well as muscle strength in their bodies. In the literature, muscle-strengthening for ankle rehabilitation has been considered with three main phases: ROM, strength, and proprioceptive training, respectively, and they require different control methods. At the beginning of the rehabilitation, the patients have limited ankle mobility; thus, passive ROM exercises are required (the patient is passive and the device is fully active) so that ankle ROM can be recovered via full assistance of the device. Then, active ROM exercise is utilized by reducing the level of assistance from device, and the patient should put effort to initiate the motion against the device. The second phase is about improving the ankle stability; as the patient's response improves, the resistance level of these strengthening activities increases. In most cases, they are unable to exert enough force to complete the exercises, so the robot should assist them. In the final phase, proprioceptive training should be performed to improve the balance control (Bernhardt et al., 2005; Valles et al., 2017; Teramae et al., 2018). However, due to their limited design configuration, that is, low weight-bearing capacity or small end effector area, most rehabilitation platforms cannot be used in the balance rehabilitation; the detailed description is given in Section 2.3. The control strategies in the stated phases are also illustrated in Table 1.

**Table 1:** Control methods in rehabilitation (Saglia et al., 2010).

**Exercise method**	**Patient mode**	**Control method**
ROM	Passive	Position control
	Active	Assistive control
Strength training	Active- isometric	Position control
	Active- isotonic	Admittance control
Proprioceptive training	Active	Hybrid control

Even though robotic-assisted rehabilitation has a promising effect on the treatment itself, especially with the capability of being repetitive and task-specific, implementation of these potentials at the desired level is still challenging. There are numerous methods for control of these robots, yet control-related challenges are to be addressed (Marchal-Crespo and Reinkensmeyer, 2009; Dzahir and Yamamoto, 2014; Li et al., 2017; Jin et al., 2018; Dong et al., 2021). Position control is one of the most used methods due to its simplicity. In this approach, the robot tries to follow a predefined motion trajectory (Mohebbi, 2020). Furthermore, the position control is needed to conduct the movements in the first phase along a specific trajectory at a constant speed to improve the ROM capacity of the patient (Girone et al., 1999; Saglia et al., 2013; Zhang et al., 2016; Ayas and Altas, 2017). However, this method cannot be used in the other phases since when the patient applies more than the expected force/torque during rehabilitation, the position method cannot compensate for this disruptive effect, which leads to excessive position tracking errors. Despite this, it should be noted that most of the existing interaction controllers continue to use a position control scheme as an inner control loop, with a corresponding force/torque or motion outer loop applied to complete the interaction controller (Lu et al., 2016; Song et al., 2019).

To eliminate the problem with position control, impedance control, which provides the desired dynamic interaction between the robot and its environment, is proposed. The physical interaction is expressed as the dynamic relationship between the motion variables of the manipulators and the contact forces that need to be kept within a predetermined safe or acceptable position trajectory while the robot follows the desired motion trajectory (Song et al., 2019). Admittance control uses force as an input and displacement as an output, while the robot follows predetermined force. These methods are used in the strength phase to assist/resist the patients (Saglia et al., 2013; Ibarra and Siqueira, 2015; Sun et al., 2015; Jamwal et al., 2016). However, these methods require force/torque sensor to measure the force exerted by patients, which adds extra complexity, that is, sensor dynamics, cost, and computational load. Moreover, the estimation of impedance parameters peculiar to the individual is another challenge (Codourey, 2016).

The dynamic model of robots is usually composed of non-linear functions of the state variables (joints' positions and velocities), especially in parallel structures. This characteristic of the dynamic model makes the closed-loop control system non-linear and difficult to solve. The computed torque control (CTC) method requires a good knowledge of the robot dynamics since the dynamic model of the manipulator is used in the loop. Even though CTC can compensate for non-linearity since dynamic equations are solved in real time, it creates a computational burden (Tsoi et al., 2009; Asgari and Ardestani, 2015; Codourey, 2016).

The above-mentioned issues are addressed by using the parameter estimation control method. With this method, the simplification of complex mathematical dynamic equations and modeling of unmodeled noise signals are done and a new physical model with functional properties can be obtained with less computation time and acceptable control performance. Furthermore, modeling can be done online or offline. In the offline method, if the estimated model is not highly accurate, it cannot be able to correctly distinguish between responses caused by known and unknown input signals. In the online method, parameter estimation is done simultaneously within the process, causing a delay in the system response (Wolbrecht et al., 2008; Gao et al., 2014; Song et al., 2019). The fuzzy logic method is also used in the control of rehabilitation robots. Furthermore, it provides good performance for the control of the non-linear system. Fuzzy logic is similar to human thought systematic than traditional logic systems. Basically, it tries to capture the approximate, imprecise nature of the real world. It has three steps: fuzzification, linguistic rules, and defuzzification. In the fuzzification step, input signals are converted into fuzzy sets with some degree of membership (range from 0 to 1). The main part of fuzzy logic control is controlling the robot using a set of linguistic control rules associated with binary concepts such as fuzzy inference and computational inference rules. In the defuzzification, fuzzy truth values are converted into output decision values. However, like parameter estimation, it is not robust against unexpected situations during real-time control since its rules are determined previously (Karasakal et al., 2005; Lamamra et al., 2020; Sharma and Obaid, 2020).

In the above-mentioned methods, the patient passively follows the movements of the robot, which follows the previously specified position or force/torque references (Chiaverini and Sciavicco, 1993; Patarinski and Botev, 1993). Recent studies in robotic therapy show that continuous passive training therapy does not significantly improve motor function. Active participation of patients is considered to be a major factor contributing to the neural plasticity and motor recovery (Keller et al., 2013; Teramae et al., 2018). One of the most commonly adopted assistance strategies, the “Assist-as-Needed (AAN)” paradigm, offers the mode of necessary active assistance to stimulate neuroplasticity. The basic principle in AAN is to provide physical assistance only when needed by the patient. If a patient performs a task flawlessly, the robotic assistance is withdrawn. However, if the patient has difficulty or cannot complete the task, the robot provides as much support as the patient needs to perform the task (Pehlivan et al., 2017). In other words, AAN is a strategy of regulating auxiliary forces/torques or task difficulty according to patients' disability level or performance in training tasks. There is strong evidence that active participation induces neural plasticity, and therefore controllers should intervene minimally to promote participation and recovery. In addition, upper extremity rehabilitation using the AAN paradigm is shown to be the most promising technique for promoting recovery (Luo et al., 2019).

New technological devices and methods are being developed to increase active patient participation by integrating multi-sensory information and allowing AAN paradigm. However, the implementation of AAN paradigm is still challenging, since determining the level of assistance according to the patient progress is not to be addressed sufficiently.

### 2.3 Design of existing rehabilitation robots

Considering the design and development of robotic devices for lower extremity rehabilitation, there are mainly three types of system, that is, wearable exoskeleton system, ankle platform, and balance rehabilitation platform are proposed (Deng et al., 2018; Ersoy and Hocaoglu, 2023). Several examples of exoskeleton devices aim to increase the capacity of the lower limbs or reducing user effort (Pratt et al., 2004; Ferris et al., 2006; Dollar and Herr, 2008; Mooney et al., 2014). Systems such as AKROD (Weinberg et al., 2007), BioMot project (Bacek et al., 2017), KNEXO (Beyl et al., 2009), Lokomat (Jezernik et al., 2003), LOPES (Van Der Kooij et al., 2006), MIRAD project (Mir, 2022), and REX (Rex, 2022) are used to support individuals with muscle weakness in ADL. However, since the mechanisms underlying human movements and how the designed devices should interact with humans are not fully understood, there is no device that can effectively improve the user's performance. The mentioned systems have difficulties in use because they have a rigid, bulky structure, and uncomfortable interfaces, restrict biological joints, and are misaligned with natural joints. In addition, if the exoskeleton does not have enough degree of freedom (DoF) to work in harmony with human joints, it exerts a residual force on the human limb due to axial misalignment, and this may cause long-term injuries, as well as discomfort (Schiele, 2008, 2009).

Platform-based robots are grounded and have movable end effector as a rehabilitation platform with one or more DoF. These types of systems employed for the ankle joint focus only on improving the ROM of the joint rather than improving the balance of the patient (Díaz et al., 2011; Morris et al., 2011). Most of the platforms for the ankle joint are in parallel structure, which provides sufficiently high torque for plantar flexion/dorsiflexion, inversion/eversion, and adduction/abduction movements of the ankle (Chablat and Wenger, 1998; Rastegarpanah et al., 2016). There are also systems designed to be serially connected to each other with motor-operated joints (Saglia et al., 2019). Although serial manipulators are easier to model, using a parallel manipulator in such an application provides advantages in terms of achieving high load-carrying capability, better dynamic performance, and precise positioning. Rutgers ankle is a Stewart platform-type haptic interface that provides 6 DoF resistance forces to the patient's foot in response to VR-based exercises (Girone et al., 2001). Various clinical studies are conducted with this device showing improvement in patient strength and endurance measurements (Girone et al., 2000; Deutsch et al., 2001; Cioi et al., 2011; Deuschl et al., 2020). In RePAiR, a 1-DoF robotic platform allows dorsiflexion and plantar flexion movements to patients after stroke. It is demonstrated that the device provides benefits in increasing the muscle strength of the patients, improving the motor control, sensory-motor coordination of the patients, and, accordingly, the walking patterns (Gon calves et al., 2014). Only a few of the manipulators developed for ankle rehabilitation are commercialized (Saglia et al., 2013; Bre, 2022; Opt, 2022). These platforms are widely used to strengthen ankle joint movements and improve ankle proprioception. The end effector of the above-mentioned manipulators are only one foot large and have a low weight-bearing capacity; therefore, they cannot be used for balance rehabilitation after ankle treatment has been completed. In addition, the end effector of the proposed systems are not endowed with a sensor; therefore, pressure change measurement on the sole and sensory input under the foot cannot be performed during the ROM rehabilitation.

Posturography, measurement of CoM and balance variables, is tested through static or dynamic techniques for balance evaluation and rehabilitation (Prosperini and Pozzilli, 2013; Park and Lee, 2014). Training with static balance platforms is said to be helpful in controlling pressure distribution in patients over time. Static posturography is usually done by using Wii fit (Wii, 2021) board or commercially available force platforms (AMT, 2022; Ber, 2022; HUR, 2022; Kis, 2022). Studies evaluated the Wii exercise experience of the patients and their physiotherapists, are stated that Wii exercise is an amusing and challenging way to improve balance impairment since VR provides continuous feedback (Prosperini and Pozzilli, 2013; Plow and Finlayson, 2014). Another study was conducted for 6 week with commercially available force platform and virtual reality and the result shows that the CoM point deviation decreased. However, when compared to static situations, rehabilitation under dynamic conditions contributes more to the improvement of balance disorders and motor skills (Prosperini and Pozzilli, 2013).

Dynamic platforms require instantaneous dynamic movements, forcing patients to adjust their balance during perturbation (Prosperini and Pozzilli, 2013). A study shows that dynamic strength platforms are more helpful in restoring postural stability than conventional therapy (Saglia et al., 2019). Wooden balance platforms are one of the simplest examples, but they do not provide any quantitative measurement (Bal, 2021). The Bobo Balance platform provides force measurement on top of wooden platforms. However, it is difficult to use for people with severe loss of balance since there is no external support environment or mechanisms for patient while standing (Bobo, 2021). gePRO (Geapro, 2021), BackinAction (Ria, 2022), Balanceback (Bal, 2022), and Proprio (Pro, 2022) platforms have 2-DoF (roll and pitch) for balance rehabilitation. These systems deliberately put patients in an unbalanced state while patient following the VR game, thus assessing their balance status based on CoM position. During the dynamic rehabilitation process, the angle of the platform can be controlled, and patients are requested to maintain their CoM and posture. Even though ROM can be covered fully by gradually changing the angular position, these systems cannot provide AAN paradigm (even if a patient performance improves, the system's level of assistance remains unchanged) (Marchal-Crespo and Reinkensmeyer, 2009; Kang et al., 2015). Furthermore, weight transfer in balance training cannot be provided during PBT due to the lack of sensory input to the foot with these devices. Additionally, the VR environment is the only sensory input proposed with these devices; however, it has been stated in the literature that multi-sensory input is more powerful for mimicking and enhancing ADL (Sigrist et al., 2013).

Investigation of APAs and CPAs, which is necessary for proactive and reactive balance control, is claimed to disclose essential aspects of postural control and history of falls. The studies show severe APA and CPA deficiencies after evaluating the patient's condition and muscular activity; however, retraining APAs and CPAs is possible through PBT programs. Despite the benefits of these studies, to our best of knowledge, there is no study in the literature that assesses reaction time, CoM, CoP, and the ability to control APAs and CPAs on ankle, balance, and stepping rehabilitation simultaneously. On top of that, the effects of multi-sensory inputs and cognitive control strategies suitable for the use of patients with different severity levels have not been evaluated and utilized.

## 3 Results

The ability of balance is a multivariate concept that integrates both motor and sensory components. Postural control during standing and walking requires multi-modal sensory feedback, for example, visual, vestibular, and proprioceptive feedback (Horak, 2006). Sensory inputs have contributions depending on the environment and the motor task performed by the individual, and patients with neurological damage live difficulties in weighing and utilizing sensory inputs (Negahban et al., 2011; Costantino et al., 2017).

Rehabilitation of balance should be performed with the integration of ankle-foot, balance, and stepping phases to enhance activity-based neuroplasticity. Therefore in this study, we propose an I-BaR framework that adopts AAN paradigm for balance analysis, rehabilitation, and assistance with multi-modal feedforward and feedback signals.

It is known that the sensory input training can positively affect motor control during balance rehabilitation (Rossignol et al., 2006; Bottaro, 2008; Laaksonen et al., 2012; Carey et al., 2016). Multi-modal information provides certain advantages in terms of effective and realistic training to mimic ADL. For instance, the ability of visualization shows spatial aspects, while audio/tactile feedback allows temporal aspects. Studies on the neurophysiology of somatosensory information processing emphasize that multiple cortical and subcortical brain (CNS and PNS) structures take an active role in sensory discrimination tests (Sigrist et al., 2013; Lee et al., 2018; Pan et al., 2019). Moreover, a stimulators should be placed on the skin of the patient so that muscles with low activity in sEMG measurement can be triggered. VR environment should be employed to improve the effectiveness of the training and patient engagement while serving as visual and auditory feedback. VR tasks should be designed similarly to the activities that the patient has difficulty and their difficulty levels should be adjustable.

**Figure 1:**
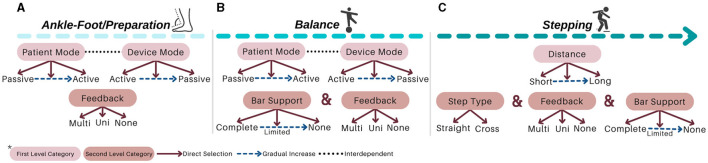
Protocol of I-BaR: **(A)** ankle-foot/preparation, **(B)** balance, and **(C)** stepping phases.

**Figure 2:**
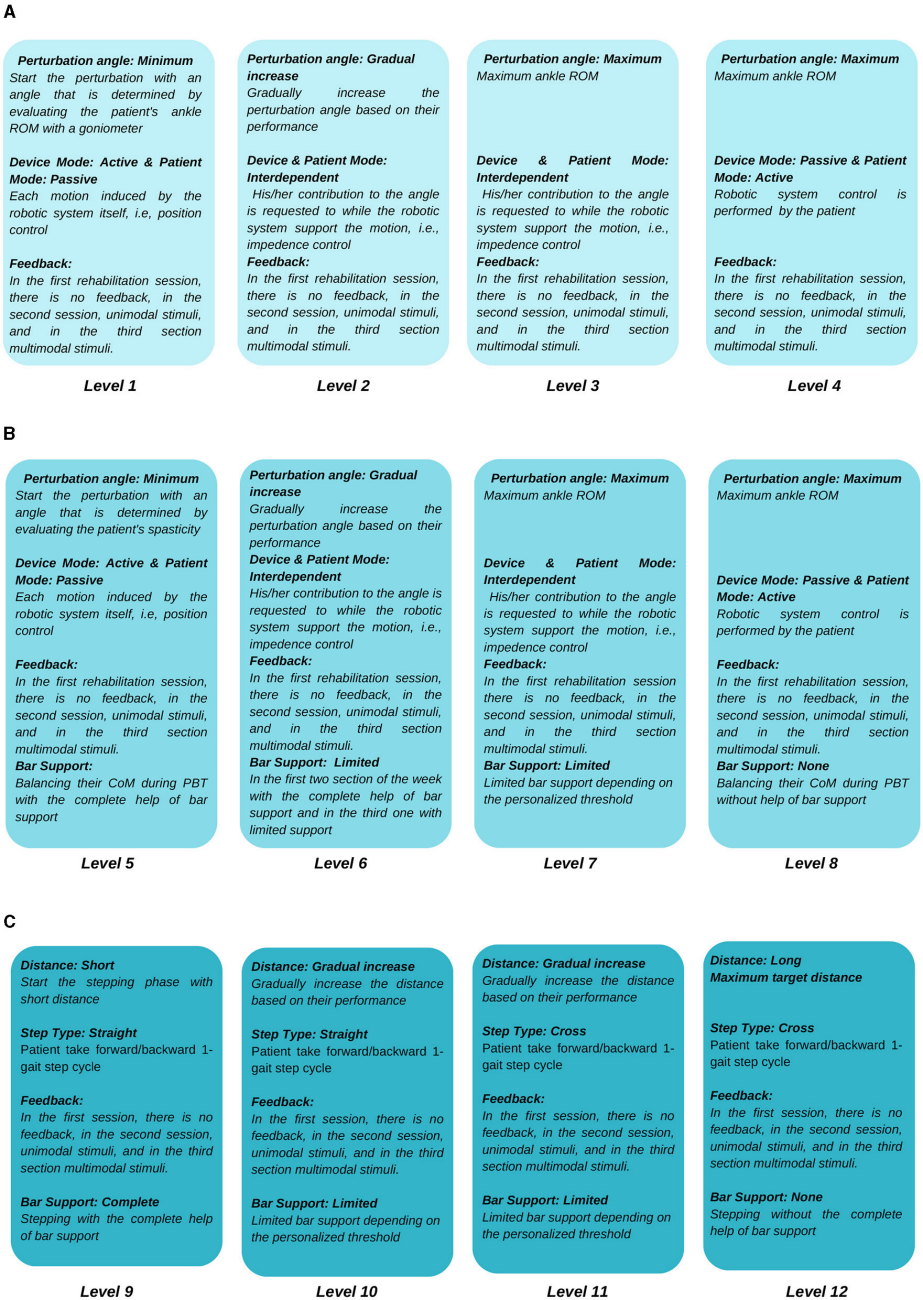
Protocol of I-BaR: **(A)** ankle-foot/preparation, **(B)** balance, and **(C)** stepping phases.

I-BaR framework proposes a personalized approach that allows patient-specific difficulty levels in three main phases of rehabilitation (see Figures 1, 2). In ankle-foot phase, after the mode of the patient (see Figures 1A, 2A) is determined and accordingly the selected robotic device should be controlled with the AAN paradigm, feedback mode can be selected according to the patient's needs. Similarly, for the balance phase (see Figures 1B, 2B), personalized rehabilitation can be offered according to the patient's capability by selecting different combinations from the bar support and feedback mode section. Moreover, after the distance is determined in stepping rehabilitation (see Figures 1C, 2C), rehabilitation can be done with the combinations of the feedback mode and step type sections mode. In this way, rehabilitation can be provided to the patient at different severity levels in the areas they completely lack. Since each option will be increased gradually throughout the process, he/she can continue their treatment without difficulty.

### 3.1 Ankle-foot/preparation phase

**Figure 3:**
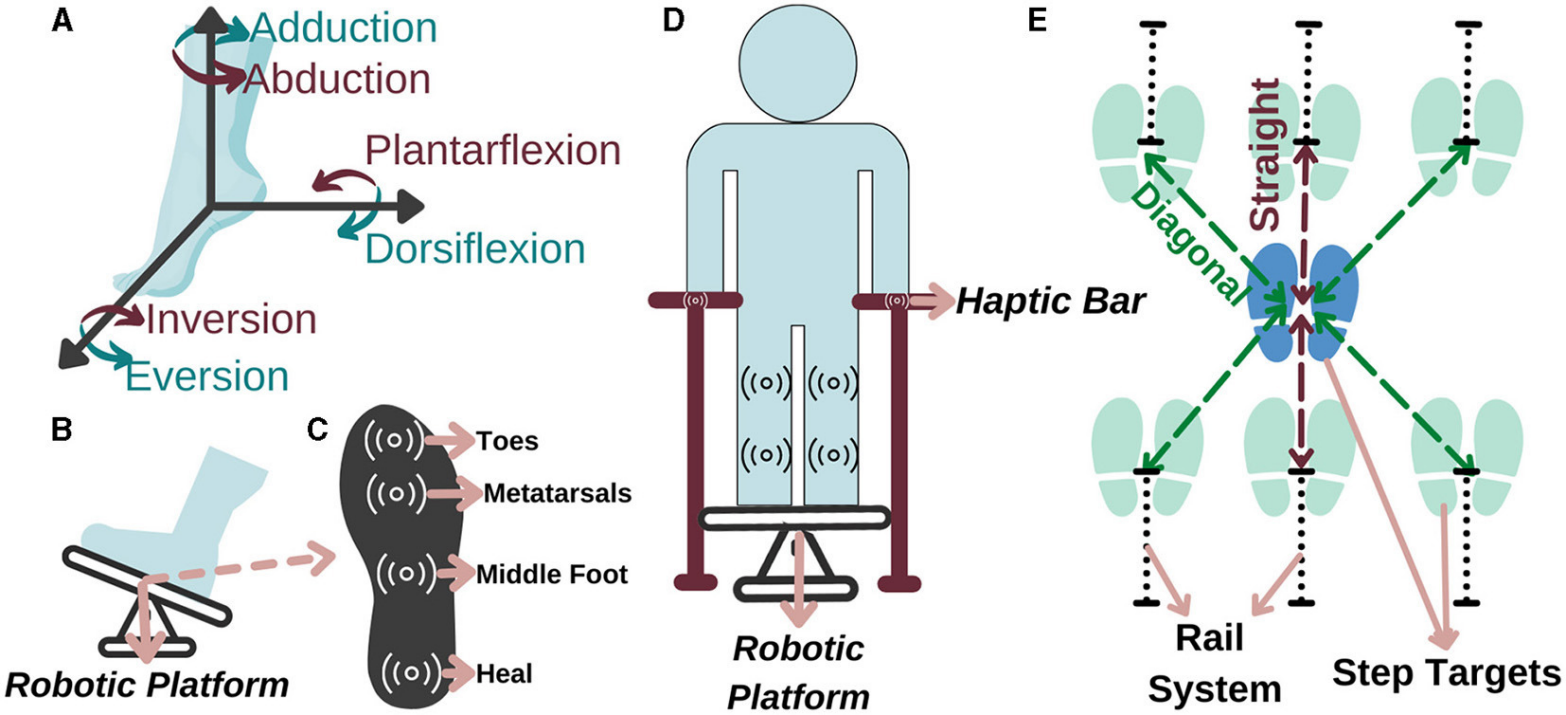
Representation of **(A)** the ankle DoF, **(B)** ankle-foot rehabilitation, **(C)** sole of the foot, **(D)** balance, and **(E)** stepping rehabilitation.

The first phase is ankle-foot/preparation rehabilitation to improve lower extremity muscles, while the patient is sitting due to their ankle instability and low muscle activation. It aims to prepare the sensorimotor system for motor function, which is essential when there is minimal or no voluntary motor/muscle function in ankle-foot. The selected robotic platform should be kept in ankle ROM, shown in Figure 3A with 0–20^*o*^ dorsiflexion, 0–50^*o*^ plantarflexion, 0–10^*o*^ adduction, 0–5^*o*^ abduction, 0–20^*o*^ eversion, and 0–35^*o*^ inversion limits (Hasan and Dhingra, 2020). The system needs to ensure that each patient can perform exercises at their specific ankle ROM limits. Upper limits must be determined according to the impairment levels of the patient, and it should be gradually increased based on their performance. Such limits can be identified with the use of a robotic platform with a predetermined force/torque based on an individual's parameters, that is, foot mass, size, and inertial parameters. This torque should have the maximum magnitude to be able to move the individual's foot. Patient can exert reaction force/torque due to spasticity (involuntary muscle contraction) and RoM limits during robotic platform induced motion. Thus, once platform cannot move the foot any further, this angle is considered as patient's spasticity level. Up until this time rehabilitation is performed with patient passive mode; however, as patient progresses in the therapy, his/her contribution to the motion would also be requested at this phase (patient active mode). For instance, moving the ankle to 5^*o*^ of dorsiflexion with patient active contribution while the platform supports and this support can be regulated according to his/her progress.

Accordingly, in a study conducted with 459 people after a stroke, it is observed that 45% of the patients had a decrease in lower extremity sensation (Sarah et al., 2013). Therefore, a force and haptic feedback sensor should be placed in at least four regions, namely, toes, metatarsals, middle foot, and heel, as they are more intense regions (Perttunen, 2002) (see Figure 3B for feet placement and see Figure 3C for specified regions). With the force sensor, the pressure applied by the patient to the each region on the sole can be measured and if the pressure distribution exceeds the able-bodied pressure distribution value in one of these regions, he/she can be stimulated that region via haptic feedback based on the individually identified sensing threshold. Accordingly, the patient can be aware of their amount of weight-bearing and sole pressure; thus, they can be trained to transfer their weight correctly for fall prevention. With this aim, a personalized preparation therapy program is proposed as an essential stage for I-BaR, which provides multi-modal feedback/feedforward signal and determines the somatosensory level of patient for the therapist to plan and decide on the parameters of the next phase.

### 3.2 Balance phase

The second phase is balance rehabilitation to improve postural adjustments while the patient is standing on the platform. Although the patient pass to this phase after the preparation, they may have difficulty in balancing their posture without physical assistance. Therefore, a haptic bar should be installed on the selected rehabilitation system (see Figure 3D). This haptic bar should at least include two sensors, force and haptic sensors, to quantitatively measure applied force and give haptic feedback accordingly. In the case of a patient with higher balance loss, the phase should be executed with full assistance of haptic bars. Throughout the rehabilitation as the patient's postural control improves, an upper extremity support limit value should be established for each patient. When the patient goes over the limit, haptic feedback is applied to warn them to reduce the upper extremity support and help them to control the posture by using the lower extremity rather than relying on the arms.

As the core part of balance rehabilitation, this phase should be utilized to increase sensory and motor integration during task-specific activities. Patient-specific information, such as ROM and proprioception, obtained from the preparation phase determine the required parameters of the therapy at this phase. This rehabilitation can also increase somatosensation during task-specific activity. Within the scope of these interventions, the patient's balance in the mediolateral and anteroposterior directions can be disrupted by giving sudden and fast perturbations with rehabilitation robot. In PBT, to train balance reactions, perturbations in different directions and amplitudes are applied, and various scenarios are used, including simultaneous cognitive and motor activities. The objective of this is to decrease the reaction time and deviation in CoM and also to improve pressure distribution as well as APAs and CPAs (Mansfield et al., 2015; Jagdhane et al., 2016). As in the preparation phase, the AAN control paradigm should be implemented on the Robotic platform system during PBT so that the patient can engage with the dynamics of the platform independently and actively within the limits of their capacity.

### 3.3 Stepping phase

The third phase is stepping rehabilitation to improve walking parameters, that is, the stance and swing phase's temporal and spatial parameters. While step speed is increasing, improving the stepping accuracy is another target in this phase. It should include assessment and training of stepping to various target distances with robotic help. Step-taking activities are performed to assess the effectiveness of the previous two phases by analyzing the relationship between muscle activity, postural control, movement speed, and accuracy as well as to train walking parameter with Fitts' law. Particularly, at stepping long distances, the patient realize the movement faster with their own compensation strategies which reduces the quality of motion, whereas at stepping short distances their motion is slower, controlled and precise. Current step-taking assessments are based on manual change of the target distance and reaction time measurements (detected by a switch button according to the time between target point and initial position force detection) (Aloraini et al., 2019, 2020; Yamada and Shinya, 2021). Since force measurements and haptic sensors are not implemented at these target points, a kinetic evaluation and improvement in sensory input under the foot cannot be provided. Therefore, in the stepping phase, the distance of the target points should be automatically adjustable within the system to provide perturbation/moving target in x-y plane and these target points should include force and haptic sensors to calculate GRF and increase sensation under the sole, respectively (see Figure 3E).

This study proposes a completely personalized rehabilitation system for patients with different severity levels or disorders. It integrates three main phases of the I-BaR framework, that is, ankle-foot, balance, and stepping rehabilitation. The originality of the study includes the following points:

- In the ankle phase, an individual preparation therapy program can be provided by identifying individuals' somatosensory disorders and providing the sensory information they need.
- In the balance phase, both static posture, proactive balance and reaction time, and reactive balance evaluation and training required for postural control can be provided.
- During the stepping phase, the weight transfer capacity in the stance phase is evaluated and improved.
- A rehabilitation method that evaluates CoM, ROM, sole pressure distribution, and sub-sole sensory input parameters is recommended.
- Proprioception perception level can be measured, somatosensory disorder levels can be measured, and the rehabilitation process can be planned individually.
- In the integrated balance rehabilitation process, integrated subcutaneous sensory input via haptic feedback improves somatosensory information and weight transfer on the sole.
- It is integrated with VR games to increase the continuity of treatment and the effectiveness of treatment with sensory feedback.
- It uses multi-modal feedback for improving sensory weighting skills.

## 4 Discussion

Rehabilitation helps with the physiological and functional recovery of motor and sensory skills that have been lost (Riemenschneider et al., 2018). Recently, it has been stated that investigating the APAs and CPAs, which are required for proactive and reactive balance control, may reveal critical information about postural control and fall history. Early muscle activation and decreased CoM deviation provided vital evidence that retraining of APAs and CPAs is achievable for stroke and elderly population. The literature provides the fundamentals of the development of PBT to enhance postural control and balance in patients to prevent fall. The therapy procedure used for large body structures, such as the lower extremities and trunk, requires a lot of physical effort for therapists and patients, and the effectiveness of rehabilitation depends on the therapist's personal knowledge and experience (Kalita et al., 2021). Robotic rehabilitation studies are increased significantly to overcome these difficulties since it is recognized as efficient and provide precise assessment of kinematic and dynamic parameters as well as objective evaluations that enhance the treatments. Yet, there are still challenges to be addressed, such as limited accessibility of present robotic devices due to cost, the training of all patients at the same level of difficulty, and insufficient personalized approach (Saglia et al., 2009; Ilett et al., 2016).

To the best of our knowledge, the aforementioned limitations have been addressed in a separate manner. For instance, in ankle rehabilitation, robotic platforms are frequently used for patients with higher severity levels to improve ankle ROM, strength, and proprioceptive. Although these studies are sufficient to improve ankle instability, PBT is still required for these individuals to improve their ADL. Ankle rehabilitation robots cannot be used in balance rehabilitation because they only have the surface area to fit one foot and have a low weight capacity compared to the whole-body weight.

Moreover, in balance rehabilitation, treatment is implemented with either a static or dynamic system. Although static balance systems are useful in evaluating the patient's balance level, dynamic anteroposterior and mediolateral perturbations are required to improve plasticity in patients. Furthermore, in the dynamic balance systems, due to the lack of sensors on the end effector, these systems cannot train patients to control their weight transfer to avoid falls. In particular, the pressure change in certain regions of the foot (at least four major parts heal, middle foot, metatarsals, and toes) cannot be measured, and corresponding vibrotactile feedback cannot be applied under the foot. Accordingly, such systems are not able to improve decreased sensation on the sole.

As the third main treatment, stepping rehabilitation is implemented to improve walking parameters, stepping accuracy, and stepping ability to various target distances. Fitts' law is used to explore the role of motor planning processes. According to the law, the target's width and distance are correlated and will determine how long it takes to go quickly to a particular region. Furthermore, this law is implemented on the CSRT test to analyze the time to complete the foot-reaching task and improve the walking parameters. Various target points are placed on the environment, and the subject must step on the target accordingly to the given feedback. These target points just include a switch to determine reaction time. In other words, they are unable to assess GRF for haptic stimuli and train the patient to control pressure distribution.

It is essential to associate the aforementioned rehabilitation method with multi-modal feedback since it enables the learning of several aspects of a movement simultaneously and mimics daily life, for example, visual, pressure, audio, vibration, and proprioception. These feedback information not only promotes the development of plasticity but also provides compensation for the loss of motor function caused due to a compromised neuromuscular system. However, to the best of our knowledge, there is no study to prove the importance of multi-modal feedback in the improvement of postural adjustments and reaction time.

The three-phase framework, I-BaR, is proposed to address the aforementioned drawbacks of current rehabilitation approaches. First of all, all these three separate treatments should be implemented as a whole in a single system to improve foot/ankle functionality and postural adjustments (APAs and CPAs) for achieving high-quality walking based on objective assessment. Moreover, in all these phases, using the multi-modal feedback for improving sensory weighting skills, the AAN paradigm for modulating the level of assistance according to patients' progress and mimicking the situations that the patient encounters in ADL should be implemented as a whole for an effective assessment and rehabilitation to achieve gradual independence eventually. Therefore, the I-BaR framework offers an effective solution as a whole with these properties to achieve personalized balance rehabilitation for different disability levels.

In this context, the requirements to be considered to implement the I-BaR framework and to ensure the development of the patients can be divided into two main groups, and they are summarized in Tables 2, 3.

**Table 2:** I-BaR framework anthropomorphic requirements (limit reference for ROM: Hasan and Dhingra, 2020, PBT: Freyler et al., 2015, and Stepping: Krebs and McGibbon, 2001).

**Exercise method**	**Anthropomorphic Limit**	**Personalization**
ROM	Considering the healthy human ankle joint, the range of motion of the ankle is identified in three directions with the following ranges: 20^*o*^ dorsiflexion, 50^*o*^ plantarflexion, 10^*o*^ adduction, 5^*o*^ abduction, 20^*o*^ eversion, and 35^*o*^ inversion	Each patient maximum ROM limit should be determined accordingly to torque/force applied on the system (based on the sEMG and force sensor measurement)
PBT	Considering the healthy adult response and movement time, the average CoM control deviation is between 2–3 cm. Thus; the displacement of CoM and perturbation velocity should be 2–3 cm 0.11–0.18 m/s, respectively	Each patient perturbation speed should be arranged accordingly to the patient postural control level (based on the sEMG, force sensor measurement, CoM deviation, and haptic bar/upper extremity support)
Stepping target perturbation	Considering the healthy adult walking speed is 1.2–1.3 m/s, step target distance should be placed with proportional to one-gait cycle and targeted reaction time	Target position should be determined accordingly the second phase response (based on the sEMG, force sensor measurement, CoM deviation, and haptic bar/upper extremity support)

**Table 3:** Feedback/feedforward requirement.

**Sensor/method**	**Limit**
Haptic sensor	Threshold value should be determined accordingly to individuals EMG and weight transfer capacity
Force sensor	Weight capacity should be at least 100 kg to cover so that 95% of the user population would be compensated.
Position control	Should be used to prepare the sensorimotor system for motor function when there is minimal or no voluntary motor/muscle function in ankle-foot.
Impedance control	Should be used to adjust patient's spasticity level (ability of force application)
AAN paradigm	Should be implemented in the control structure to provide physical assistance only when needed by the patient and thus to stimulate neuroplasticity
Feedback type	Multi-modal sensory feedback (Audio, haptic visual, vestibular, and proprioceptive) should be implemented on the rehabilitation procedure to integrates both motor and sensory components as whole and thus improve ADL.

Physical system requirements;

- At least 3-DoF [roll (rotation in x), pitch (rotation in y), and (elevation in height (translational in z)] to mimic ADL.
- System should be able to determine applied force/torque based on an individual's parameters (Patient's ROM, ankle torque) to keep the treatment at the limit that the patient can have difficulty continuously.
- AAN paradigm implementation on control structure to encourage active participation of the patient.
- System's end effector should include at least four force and haptic sensors on each foot (should cover at least the toes, metatarsals, middle foot, and heel) to quantitatively measure applied force and give haptic feedback accordingly.
- Haptic bar should be installed on the system to provide physical assistance on balance and stepping rehabilitation.
- Haptic bar should be include force and haptic sensors to measure upper extremity support and this support should be reduced by haptic feedback (if the patient depends to upper extremity to walk or control the posture).
- Target point on the stepping rehabilitation should be automatically adjustable to provide perturbation/moving target in x-y plane.
- Target point on the stepping rehabilitation should be include force and haptic sensor to quantitatively measure walking parameter (GRF on stepping) and increase sole sensation.

Rehabilitation requirements;

- Muscle which has low activity should be triggered with haptic feedback to initiate the motion during all phases.
- Each patient should perform the exercises at their specific ankle ROM and torque/force to maintain the patient safety.
- Patient's balance in the mediolateral and anteroposterior directions should be disrupted by large and sudden perturbation to improve plasticity and balance reactions (neuromuscular responses).
- According to the patients at sub-threshold vibration sensing level, haptic feedback should be provided to improve decreased sole sensation.
- Multi-modal feedback (Haptic, audio, visual, physical (anteroposterior and mediolateral), and perturbation) should be used to improve sensory-integrating weighting skills.
- Ankle-foot, balance, and stepping rehabilitation should be performed as a whole, starting from the necessary phase according to the patient's initial physical condition, to achieve gradual independence.

The I-BaR framework includes servo motors/linear actuators, force and haptic sensors, a data acquisition system, EMG, and motor drivers. Furthermore, to construct this system, the average and estimated cost of the framework is calculated and presented in Table 4. It presents the details of the overall system cost, which is 26,850 euros. The cost of manual balance balls or products lacking sensors ranges from 400 euros to 2,110 euros. Integration of a virtual reality environment into these systems increases the price to a range of 5,000 euros to 10,000 euros. Additionally, there are more advanced rehabilitation systems available in the market that offer perturbation, virtual reality environments, and feedback, priced between 25,000 euros and 50,000 euros. Additionally, none of these systems include an EMG device. When excluding the expense of the EMG device from the I-BaR framework, the installation cost is reduced to 12,000 euros, positioning it as a favorable option in the market. Moreover, the I-BaR Framework introduces three rehabilitation phases aimed at enhancing functionality and cost-effectiveness.

**Table 4:** Potential expenditure to realize the proposed framework.

**Item**	**Reason for use**	**Average cost (Euro)**
Servo motors/linear actuators	Should have at least 3-DoF motion, that is, 3 servo motors/linear actuators and properties of motors should chosen as human subjects limit with a safety factor	3000
Force and Haptic Sensor	To measure applied force and give haptic feedback	450
Data Acquisition Card	Should be suitable for real-time data connection	1,200
Physical construction	To physically construct the system	5,000
Simulation license	License fees for simulations to process real-time data (1ms)	1,000
EMG	To analyze muscle activation	15,000
Servo/motor driver	To control encoder data of motors	1,200
Total		26,850

## 5 Conclusion

The interdependence of sensory, cognitive, and motor processes, as well as the need for integrated training, are increasingly being employed in therapies to increase motor skills and balance and decrease the fear of falling. According to these approaches, new technological devices and paradigms must be developed to promote the participation of a paradigm from the CNS to the PNS.

In this context, we explain the necessity of the proposed I-BaR framework, which includes:

- Ankle-foot rehabilitation: ankle-foot muscle activation, sole, joint, and movement sensations are developed while sitting,
- Balance rehabilitation: sensory weighting skills are developed from motor learning by using multi-sensory input during PBT and help gradual independent,
- Stepping rehabilitation: walking parameters are improved during step-taking activities to target points with adjustable distance.

In all the phases mentioned above, using the multi-modal feedback for improving sensory weighting skills, AAN paradigm for modulating the level of assistance according to patients' progress and mimicking the situations that the patient encounters in ADL should be implemented as a whole for an effective assessment and rehabilitation to achieve gradual independence eventually.

As a future work, the design and construction of the required robotic platform are in our ongoing study for validating and evaluating this framework on patients with neurological disorders.

## Data availability statement

The original contributions presented in the study are included in the article/supplementary material, further inquiries can be directed to the corresponding author.

## Author contributions

TE: Writing – review & editing, Writing – original draft, Visualization, Resources, Methodology, Investigation, Conceptualization. PK: Writing – review & editing, Writing – original draft, Methodology, Investigation, Conceptualization. EH: Writing – review & editing, Writing – original draft, Methodology, Investigation, Conceptualization. RU: Writing – review & editing, Writing – original draft, Methodology, Investigation, Conceptualization.
